# The "F" in SAFE: Reliability of assessing clean faces for trachoma control in the field

**DOI:** 10.1371/journal.pntd.0006019

**Published:** 2017-11-30

**Authors:** Sheila K. West, Derick Ansah, Beatriz Munoz, Nicodemus Funga, Harran Mkocha

**Affiliations:** 1 Dana Center for Preventive Ophthalmology, Johns Hopkins University, Baltimore, Maryland, United States of America; 2 Kongwa Trachoma Project, Kongwa, Tanzania; RTI International, UNITED REPUBLIC OF TANZANIA

## Abstract

**Background:**

Although facial cleanliness is part of the SAFE strategy for trachoma there is controversy over the reliability of measuring a clean face. A child’s face with no ocular and nasal discharge is clean and the endpoint of interest, regardless of the number of times it must be washed to achieve that endpoint. The issue of reliability rests on the reproducibility of graders to assess a clean face. We report the reproducibility of assessing a clean face in a field trial in Kongwa, Tanzania.

**Methods/Findings:**

Seven graders were trained to assess the presence and absence of nasal and ocular discharge on children’s faces. Sixty children ages 1–7 years were recruited from a community and evaluated independently by seven graders, once and again about 50 minutes later. Intra-and inter-observer variation was calculated using unweighted kappa statistics. The average intra-observer agreement was kappa = 0.72, and the average inter-observer agreement was kappa = 0.78.

**Conclusions:**

Intra-observer and inter-observer agreement was substantial for the assessment of clean faces using trained Tanzania staff who represent a variety of educational backgrounds. As long as training is provided, the estimate of clean faces in children should be reliable, and reflect the effort of families to keep ocular and nasal discharge off the faces. These data suggest assessment of clean faces could be added to trachoma surveys, which already measure environmental improvements, in districts.

## Author summary

The repeated infection that causes trachoma is spread from person to person via infected ocular and nasal secretions. The World Health Organization (WHO) strategy for trachoma control includes keeping children’s faces clean of discharges, yet “clean faces” are not measured in most of the district surveys that evaluate the impact of programs. We studied the reliability of assessing “clean faces” in 60 children in a village in Tanzania, using trained Tanzania staff with a variety of educational backgrounds. When assessing the same child twice after 50 minutes, the staff showed substantial agreement with themselves. The agreement between a senior grader and each staff member was also excellent. The data suggest that clean faces in children can be reliably assessed, and should be added to district surveys that are measuring the impact of programs on control of trachoma.

## Introduction

Trachoma, the leading infectious cause of blindness world-wide, is caused by repeated episodes of ocular infection with *C*. *trachomatis*[1]. There is no animal reservoir, nor required intermediate host or vector, for trachoma which is spread among persons from contact with infected ocular secretions. Although *C*. *trachomatis* is an obligate intracellular organism, there is no doubt that ocular secretions contain infectious particles as the elementary body stage of the life cycle is extracellular and swabs of eyes that contain no conjunctival cells still can contain chlamydia DNA[2,3].

Ocular secretions can drain down the nasolacrimal duct and cause nasal secretions to also contain infected material [4,5]. Nasal and ocular secretions which come from children with trachoma have been found to be especially attractive to secretion-seeking flies [6]. Hands, clothes, flies, and any other material in contact with infected secretions are at risk of transmitting infection to others, but it is clear that infected secretions are the source of infections.

These secretions are visible on a face that has not been wiped or washed. In the trachoma literature, an “unclean face” has been defined as a face that has presence of nasal secretions and/or ocular secretions; some investigators also add the presence of flies on the face [7–12]. Numerous studies, cross sectional and prospective, have linked unclean faces to a greater risk of trachoma [8–10,13–16]. A clinical trial, in which villages were randomized to a community-based clean faces program, found that the risk of trachoma and severe trachoma over time was less in children with who were observed to have clean faces and especially clean faces observed over multiple observations[17]. These studies provide support for the inclusion of “Facial cleanliness” as the “F” in the World Health Organization sanctioned, multifaceted SAFE program for trachoma control.

However, there has been confusion around the value of measuring a “clean face” for trachoma control programs, primarily citing an article that claims measuring a clean face is “unreliable”[12]. Since the kappa agreements were reasonable for the individual signs of a clean face in that study, the claimed lack of reliability was based on the observation that throughout the day, the children who were washed became increasingly unclean. The authors concluded that observing a clean face was not a good marker for the washing of a child’s face. However, that conclusion is missing the point: in order to avoid transmission, a face should be kept clean of ocular and nasal secretions, regardless of the number of times it must be washed in order to achieve that endpoint. The observation that some children became increasingly unclean only points to the necessity of multiple washings that are needed.

Thus, the key issue for questioning the reliability of grading a child’s face for facial cleanliness is whether or not graders can demonstrate good agreement as to when a child’s face is clean or not. We had the opportunity to assess the intra and inter reliability of seven graders of varying backgrounds to grade markers of a clean face, and clean face status in a study in children ages 7 years and younger in Kongwa, Tanzania.

## Methods

### Population

A total of 60 children ages 1–7 years were recruited as a convenience sample in Manungu village in Kongwa, Tanzania. Children were recruited by the local health worker the day before the visit, and she obtained informal consent to have the child seen the following day. Parents signed written consent the day of the survey.

### Graders

Seven different graders were involved in the study, all members of the Kongwa Trachoma project team who ranged in background from the Medical Assistant to driver. The Master trainer (MK) who has taught clean face assessment to all staff members, was selected as the standard by whom all other graders would be measured. A half hour training session on the assessment of a clean face was held, specifically defining the face area and reviewing the signs of ocular discharge, fly on the face, and nasal discharge. All signs must be observed on faces in the absence of crying, which can distort the assessment of all three signs. The child was to be examined in sunlight, but not so that the sun was directly on the face, as that can also elicit crying. The facial area was defined as the area on direct frontal examination from hairline to chin and ear to ear. The hair itself, under chin, and neck were excluded. The signs were defined as follows:

Ocular discharge: The presence on the lid margin or lid (including in the corners) of clear or cloudy fluid, or dry matter.
Fly on the face: at least one fly must land on the face in the 3 second period of observation.
Nasal Discharge: looking at the child in frontal view, the presence of wet or dry discharge that is outside the nostril. The examiner should not stare up the nostril to find discharge, but see the discharge visible outside the nares.

An initial group of five children were evaluated with all graders in discussion together to discuss agreement.

**Fig 1:**
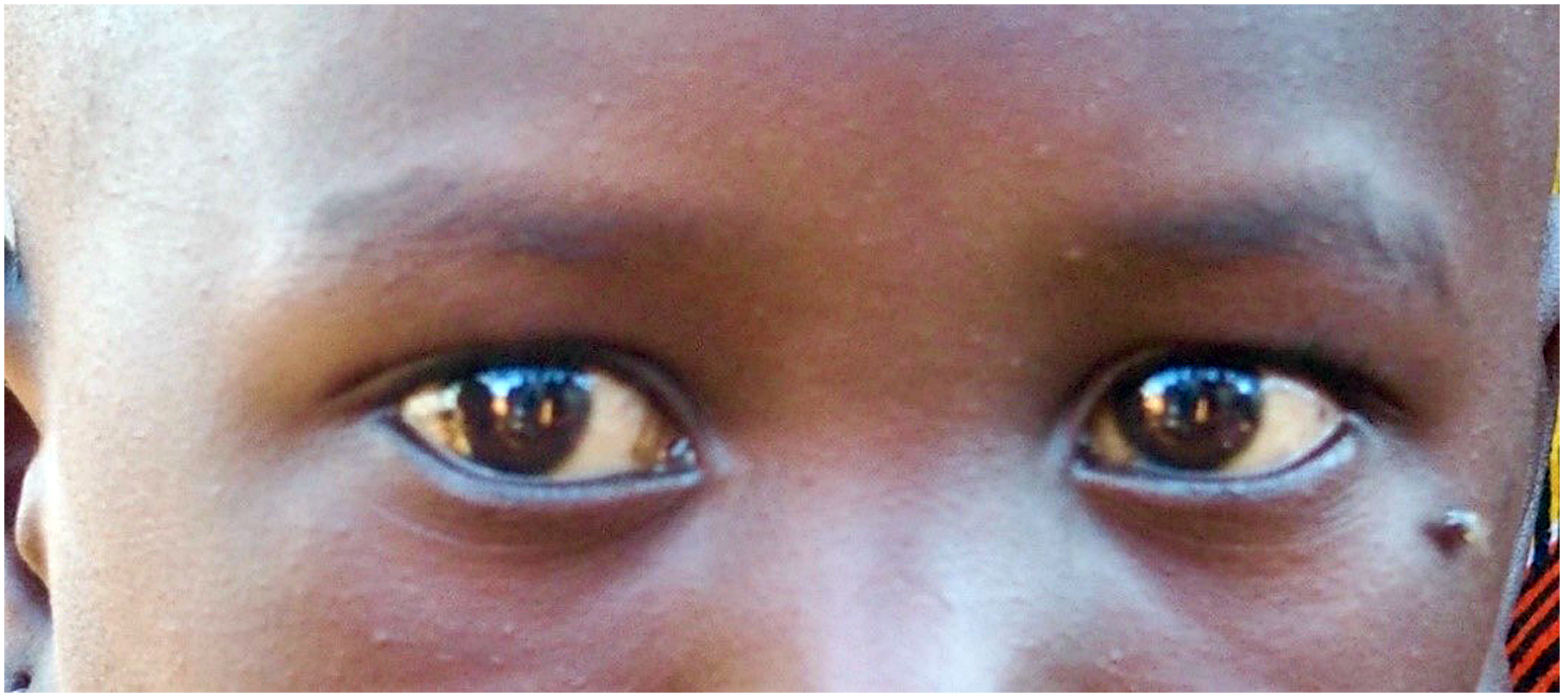
Example image of fly on face below the child’s right eye.

**Fig 2:**
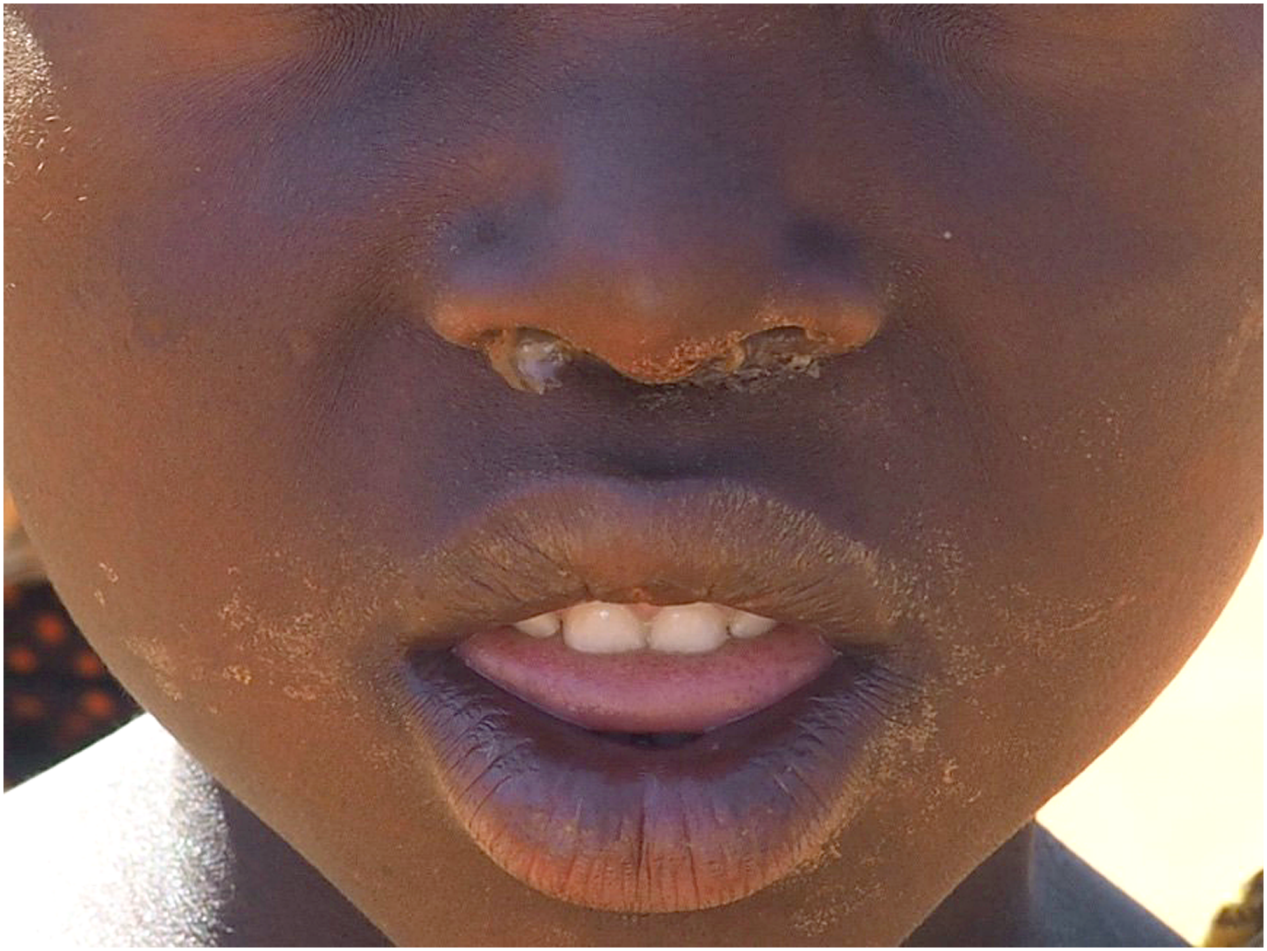
Example of a minimal amount of nasal discharge.

**Fig 3:**
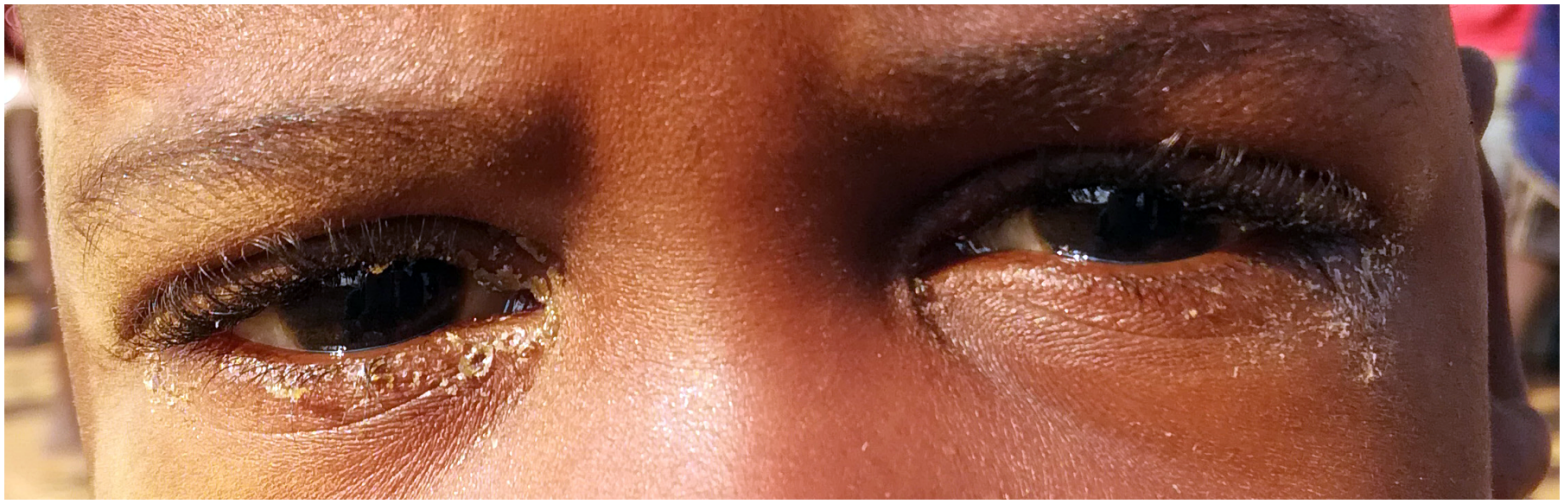
Example of ocular discharge and a crusty eyelid.

Examples of a fly on the face, the minimal amount of nasal discharge, and ocular discharge are shown in Figs 1–3. We did not capture an image of minimal ocular discharge.

After the discussion, an unclean face was defined as the presence of either ocular discharge or nasal discharge. Before the trial began, we excluded the observation of flies on the face in the definition, because during discussion, we observed that flies landing on a face was a very unstable sign and fly landings were seemingly at random and not connected to seeking eyes.

The graders were stationed at least 4 feet from one another and the 60 study children were brought to each station individually. The graders were masked to one another’s grades for both trials.

Written informed consent was obtained prior to the survey, which began at 10:15 AM. The guardians and children came to a central location, and were assigned a number from 1 to 60 which was written with a marker on the child’s hand. The graders were seated at separate stations, and each child in turn was taken to the station. After the first trial, a second trial was done 30 minutes later with the same study children. On average each grader saw the same child 50 minutes apart.

No child was dropped from the study, even if a guardian was observed cleaning a child’s face between observers, or between trials. One child did not go through the second trial, which was then based on 59 children.

### Data analyses

We calculated the prevalence of a clean face and the individual signs as determined by each grader for each trial, then determined the average prevalence as the sum of each individual prevalence divided by the number of graders. We report the range of prevalences as the lowest and highest prevalence calculated for the seven graders. We calculated the intra-observer agreement on measuring a clean face using the kappa statistic among the 7 graders who each graded the children twice after 50 minutes between trials 1 and 2. The average intra-observer agreement was calculated as the average of all the individual agreements. The inter-observer agreements were calculated comparing the senior grader (MK) against all other graders. For those grader agreements that were less than 0.7, we examined the agreement on the two individual signs to determine which one was more problematic. All data were analyzed using SAS (SAS Institute, Carey, NC). Please see S1 Dataset for all data related to this project.

### Ethics statement

This study was approved by the Johns Hopkins Ethical Review Committee and the Tanzania National Institute for Medical Research. We obtained written informed consent from the guardians of all participants, and the study was conducted according to the tenets of the Declaration of Helsinki.

## Results

In trial 1, the prevalence of clean faces as assessed by all the graders averaged 53% and varied among graders from 45% to 67% with differences driven by differences in the assessment of nasal discharge. Nasal discharge was common, average prevalence of 44%. Ocular discharge was infrequent with a prevalence of 11%, ranging from 3% to 20%. In trial two the average prevalence of clean faces declined to 50%, and varied from 42% to 58%.

**Fig 4:**
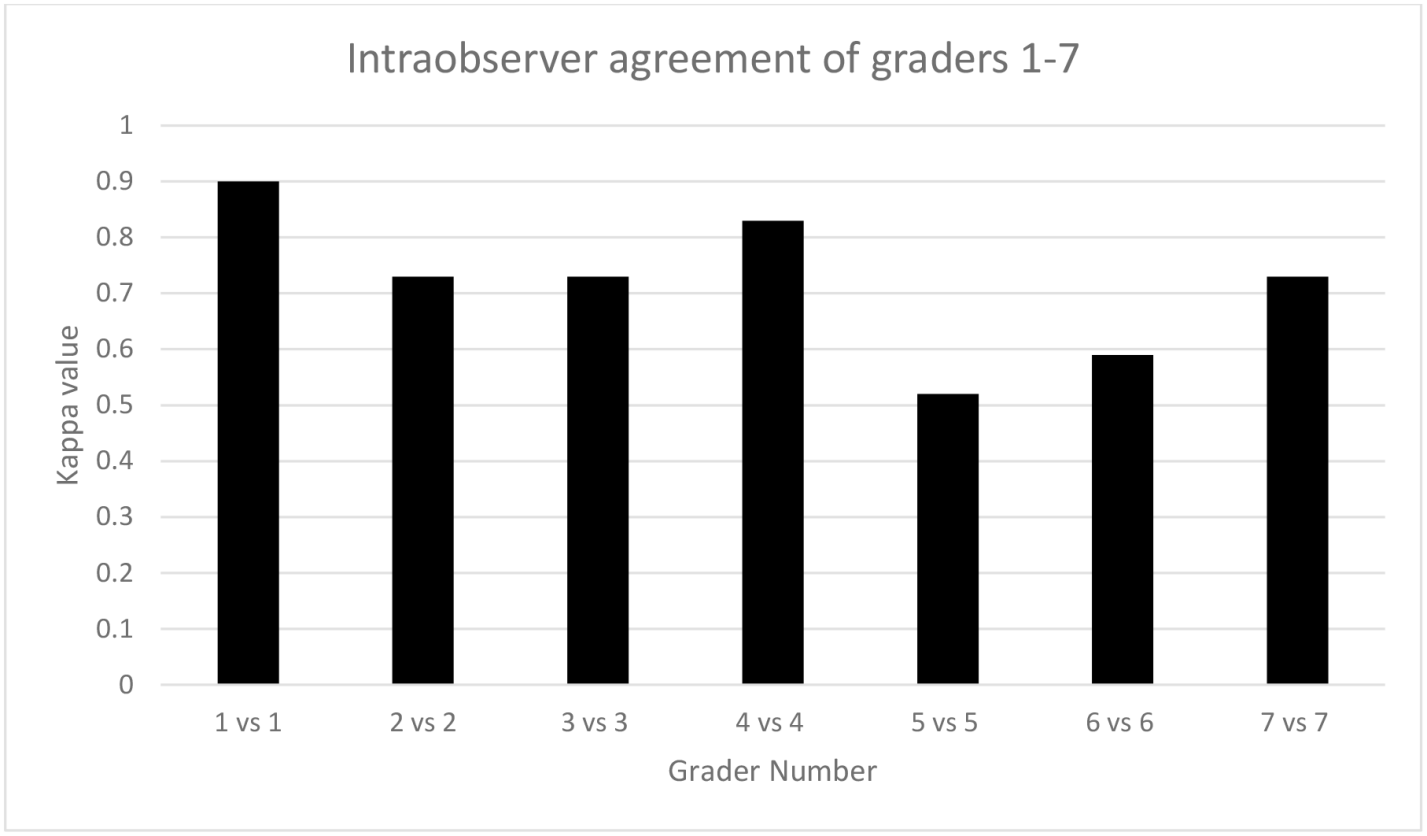
Intraobserver agreement (Kappa score) for graders 1 through 7 for assessment of clean face between trials one and two.

The intra-observer agreement based on each grader grading the child twice in trial 1 and trial 2 was good, ranging from kappa = 0.52 to kappa = 0.90 (Fig 4). Overall, the intra-observer agreement averaged kappa = 0.72.

**Fig 5:**
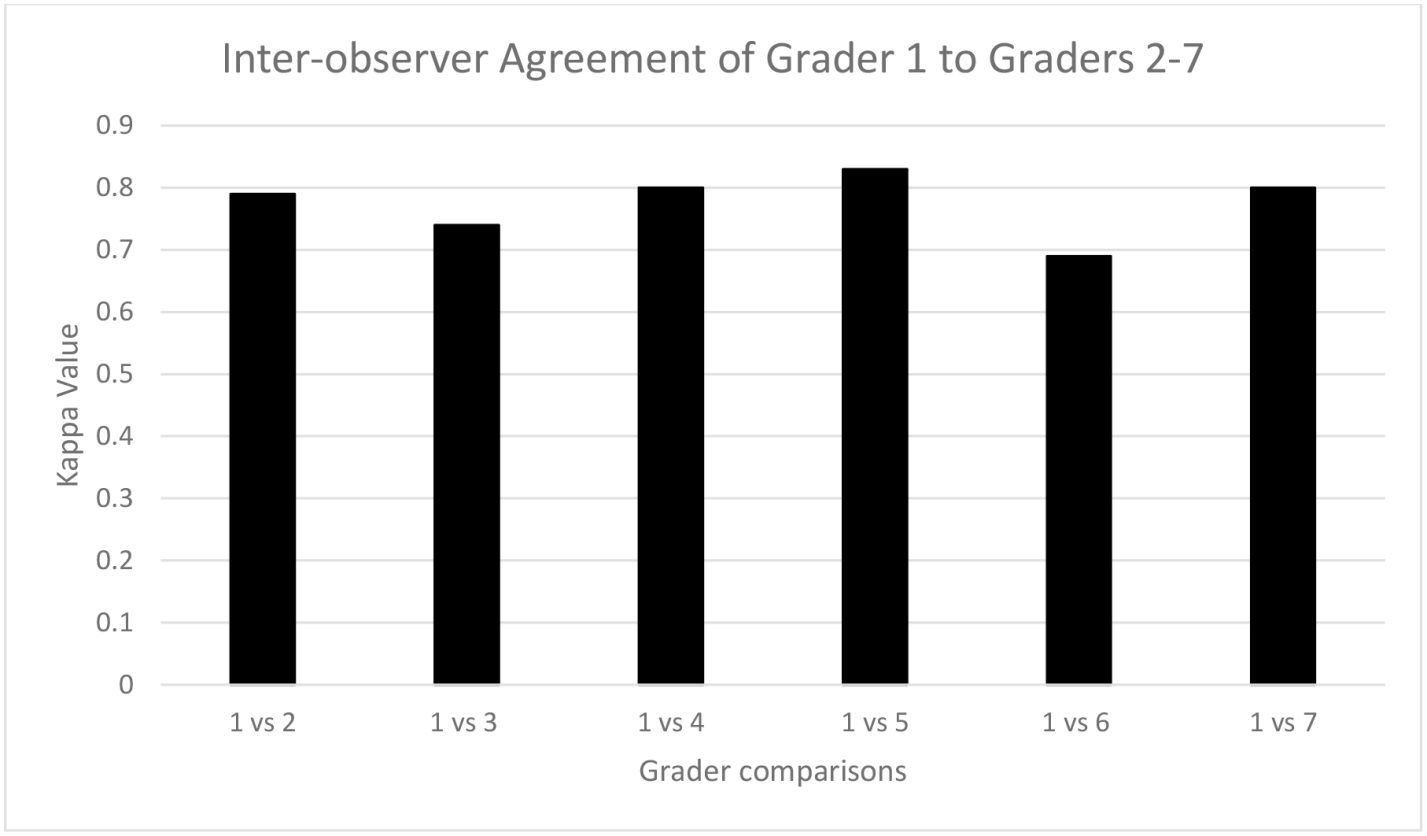
Inter-observer agreement (Kappa score) between grader 1 and graders 2 through 7.

We compared the gradings from the senior grader (MK) to the others from Trial 1. The kappas ranged from 0.69 to 0.83 (Fig 5), again with differences driven by different assessments of presence of nasal discharge. The overall average kappa was good, 0.78. Table 1a and 1b show examples of the actual agreement tables for the graders with kappas of low, 0.69, and high, 0.83, scores. There was some evidence of bias in the largest disagreement, with more disagreements where the senior grader saw more unclean faces than the other grader.

**Table 1:** Example of agreement for clean face.

		Grader 5*			Grader 6**		
		Clean	Unclean	Total	Clean	Unclean	Total
Grader 1	Clean	31	2	33	32	1	33
	Unclean	3	24	27	8	19	27
	Total	34	26	60	40	20	60

*Kappa = 0.83 (95%CI = .069–0.97)

**Kappa = 0.69 (95%CI = 0.51–0.87)

## Discussion

This study showed that the assessment of clean faces can be carried out reliably by graders with a variety of backgrounds. Regardless of whether the reliability assessment was done with a greater proportion of clean faces in the sample or a smaller proportion, only one grader had a kappa <0.7 against the senior grader.

The faces became less clean from trial 1 to trial 2, so the intra-observer agreement, while averaging >0.7, was less than the inter-observer agreement because the child’s clean face changed status over time. On average, it was 50 minutes between the time the first child started and the same child came back through.

It was interesting to note that all graders did well, regardless if they were drivers, census workers, or Medical Assistants. This suggests that training is the most important aspect for assessment of this sign, not background level of education.

One limitation of the study was the preponderance of nasal discharge driving the assessment of clean faces in this sample. The study was carried out during the season of highest prevalence of upper respiratory illness in the community, so the greater prevalence of nasal discharge was not unexpected. There was still ocular discharge as well, but the agreement on clean face in this study largely centered on the agreement in detecting nasal discharge.

We have previously shown that children assessed in the home tend to have less clean faces than when the assessment is made at a central location, so the absolute prevalence of clean faces in this sample may be higher than if assessed at the home [8]. However, that should not affect the reliability estimates.

We compared the agreement of the graders to a senior grader, to demonstrate reliability. A senior grader is not a “gold” per say, but there is no external standard of “clean face” that can be used. The designation of senior grader was based on years of experience in training graders, high internal consistency, and face validity. This approach has been used in other surveys, for example, for trachoma mapping where a Master grader (the senior scientist) was the “gold standard” by which other graders were standardized [18].

Our inter-observer reliability estimates were better than reported by King et al, who found assessment of nasal discharge to have an average kappa of 0.62 and ocular discharge of 0.46 [12]. In that study, a kappa for assessment of clean face was not done and the reliability was based on assessment of images and not on actual field assessment. There was no training program described for the multiple examiners in that study either, and the examiners were either ophthalmic nurses or ophthalmologists. Interestingly, our current results are similar to inter-observer agreements we reported 25 years ago using interviewers and an eye nurse on a sample of children in Kongwa [19].

In summary, our reliability trials found good to excellent intra-observer and inter-observer agreement for the assessment of clean faces by trained Tanzania staff who represent a variety of educational backgrounds. As long as training is provided, the estimate of clean faces in children should be reliable, and reflect the effort of families to keep ocular and nasal discharge off the faces. We recommend conducting more interobserver trials in various trachoma settings and that assessing clean faces be added to surveys for trachoma which already measure environmental improvements, in districts.

## Supporting information

S1 Dataset (XLSX)
